# Negative control of the HGF/c-MET pathway by TGF-β: a new look at the regulation of stemness in glioblastoma

**DOI:** 10.1038/s41419-017-0051-2

**Published:** 2017-12-13

**Authors:** Eleanna Papa, Michael Weller, Tobias Weiss, Elisa Ventura, Isabel Burghardt, Emese Szabó

**Affiliations:** Laboratory of Molecular Neuro-Oncology, Department of Neurology, University Hospital and University of Zurich, Zurich, 8091 Switzerland

## Abstract

Multiple target inhibition has gained considerable interest in combating drug resistance in glioblastoma, however, understanding the molecular mechanisms of crosstalk between signaling pathways and predicting responses of cancer cells to targeted interventions has remained challenging. Despite the significant role attributed to transforming growth factor (TGF)-β family and hepatocyte growth factor (HGF)/c-MET signaling in glioblastoma pathogenesis, their functional interactions have not been well characterized. Using genetic and pharmacological approaches to stimulate or antagonize the TGF-β pathway in human glioma-initiating cells (GIC), we observed that TGF-β exerts an inhibitory effect on c-MET phosphorylation. Inhibition of either mitogen-activated protein kinase (MAPK)/ extracellular signal-regulated kinase (ERK) or phosphatidylinositol 3-kinase (PI3K)/protein kinase B (PKB/AKT) signaling pathway attenuated this effect. A comparison of c-MET-driven and c-MET independent GIC models revealed that TGF-β inhibits stemness in GIC at least in part via its negative regulation of c-MET activity, suggesting that stem cell (SC) maintenance may be controlled by the balance between these two oncogenic pathways. Importantly, immunohistochemical analyses of human glioblastoma and ex vivo single-cell gene expression profiling of TGF-β and HGF confirm the negative interaction between both pathways. These novel insights into the crosstalk of two major pathogenic pathways in glioblastoma may explain some of the disappointing results when targeting either pathway alone in human glioblastoma patients and inform on potential future designs on targeted pharmacological or genetic intervention.

## Introduction

The prognosis of glioblastoma, the most common type of intrinsic malignant brain tumor, remains poor even with the current standard of care of the combination of surgery, radiotherapy, and chemotherapy^1,2^. Highly infiltrative growth patterns, cellular and molecular heterogeneity, a subpopulation of cells highly resistant to radiotherapy and chemotherapy, called glioma stem-like or glioma-initiating cells^3–6^, and a complex network of interactions between different tumor cell populations and the tumor microenvironment^7^ have been held responsible for escape from current therapies. Identification and clinical validation of new predictive biomarkers and novel multi-targeted drug combinations may have the potential to improve disease control^8^.

Among the multiple signaling pathways associated with glioblastoma, the HGF/c-MET and TGF-β pathways have gained particular attention because of their putative roles in glioblastoma SC function^9–11^, in the development of invasiveness^12,13^ and resistance to radiotherapy and chemotherapy^14–17^ as well as targeted therapies^18^.

HGF and TGF-β represent members of a large family of cytokines which are involved in the regulation of embryonic development and tissue homeostasis^8,19^. c-MET is a receptor tyrosine kinase that, after binding its ligand, HGF, activates a wide range of different cellular signaling pathways, including those involved in proliferation, motility, migration and invasion. TGF-β ligands bind and activate a heteromeric complex of type I and type II transmembrane serine/threonine kinase receptors. This complex is usually formed by ligands, activin receptor-like kinase (ALK) TGF-β type I receptors (TβR-I) and TGF-β type II receptors (TβR-II)^20^. The human genome encodes seven type I receptors (ALK1-7) and five type II receptors (ActR-IIA, ActR-IIB, BMPRII, AMHR-II and TβR-II) that pair in different combinations as receptor complexes for various members of the TGF-β family^21^. Subsequently, conformational changes in TβR-I allow the phosphorylation of signaling molecules called receptor-regulated (R)-SMAD (i.e., SMAD 2,3). The phosphorylated SMAD together with SMAD4 form transcriptional regulatory complexes. Translocating into the nucleus, they modulate the expression of many target genes^8^. Additional diversity in TGF-β signaling is achieved via activation of non-canonical, SMAD-independent pathways, including tumor necrosis factor (TNF) receptor-associated factor 4 (TRAF4), TRAF6, TGF-β-activated kinase 1 (TAK1; also known as MAP3K7), p38 mitogen-activated protein kinase (p38 MAPK), RHO (RAS homolog)-like GTPase signaling pathways, phosphoinositide 3-kinase (PI3K)—AKT (also known as protein kinase B), extracellular signal-regulated kinase (ERK), JUN N-terminal kinase (JNK) or nuclear factor-κB (NF-κB). Finally, WNT, Hedgehog (HH), NOTCH, interferon (IFN), TNF, and RAS pathways also contribute to the complexity of cellular responses to TGF-β signaling. Gene expression programs controlled by canonical and non-canonical TGF-β signaling pathways may provide tumor-suppressive or tumor-promoting functions depending on the tumor type and the stage of tumor progression^22^.

Increased c-MET and TGF-β pathway activity may promote tumor progression in glioblastoma via invasion, migration, angiogenesis, cell survival, SC maintenance, and immune evasion^13,23–28^, however, their potential interactions may not have been sufficiently studied. Here we report that these two oncogenic pathways may not act in concert, but act apparently partially antagonistic at least in human GIC models, potentially accounting for disappointing results with inhibition of either pathway in the clinic^29–31^, but also providing new concepts on how to modulate these pathways more efficiently to improve the outcome of glioblastoma.

## Materials and methods

### Reagents

EMD1214063 (c-MET tyrosine kinase inhibitor) was developed by Merck (Darmstadt, Germany)^32^. SD-208 (Scios, Inc., Sunnyvale, CA)^33^ and Galunisertib (LY2157299 monohydrate) are TβR-I (ALK-5) inhibitors (Selleckchem, Houston, TX). U0126, inhibitor of MEK1/2 (Cell Signaling Technology, CST Denvers, MA) and AZD5363, protein kinase B-alpha inhibitor (AstraZeneca, Cheshire, UK) were used. All drugs were dissolved in dimethylsulfoxide (DMSO) and diluted in cell culture medium (final solvent concentration ≤0.01%). Recombinant human HGF, TGF-β1 and TGF-β2 were from R&D Systems (Minneapolis, MN).

### Cell culture

ZH-161, ZH-305 and T-269 GIC lines were isolated from surgically removed glioblastomas^34,35^. GIC are routinely authenticated at the Leibniz Institute DSMZ-German Collection of Microorganisms and Cell Cultures, Braunschweig, Germany by short tandem repeat analysis, most recently in 2016. The cells were cultured in neurobasal medium (NBM) supplemented with 2% B27, 1% glutamine, epidermal growth factor (EGF, 10 ng/ml), and basic fibroblast growth factor (bFGF, 10 ng/ml). The cell lines are routinely evaluated for in vivo tumorigenicity in nude mice.

### Real-time PCR

cDNA generated by reverse transcription from 1 µg total RNA was used for real-time PCR (RT–PCR). Gene expression was measured using SYBR Green chemistry (AppliChem, Darmstadt, Germany) with the RT–PCR System 7300 (Applied Biosystems, Foster City, CA). All probes were tested for equal reaction efficiencies. The mean CT values for each sample were used to calculate relative expression using a variation of the 2^(- delta delta CT) method^36^. Specific target gene expression was normalized to hypoxanthine-guanine phosphoribosyltransferase 1 (*HPRT1*) and the relative expression was calculated using the formula 2^(−delta CT) (ref. 36). The RNA purity was assessed by the ratio of the absorbance at 260 and 280 nm. RNA quality was verified by the use of amplification efficiency of the reference gene (*HPRT1*).

Primer sequences are provided in Supplementary Table S1.

### Immunoblot analysis

Whole cell lysates were prepared using radio-immunoprecipitation assay (RIPA) lysis buffer (pH 7.8) containing 25 mM Tris-HCl, 120 mM NaCl, 5 mM EDTA and 0.5% NP-40 supplemented with 100 μg/mL phenylcmethylsulfonyl fluoride, 200 mM sodium orthovanadate, 0.5 M NaF, protease inhibitor cocktail sets III and IV and phosphatase inhibitor cocktails 2 and 3 (Sigma-Aldrich). Equal protein concentrations (25 μg) were loaded and electrophoresis was performed on SDS-PAGE (8–10%) under reducing conditions. Respective primary antibodies were purchased from Cell Signaling Technology, except for anti-phospho-SMAD3 (Abcam, Cambridge, UK), anti-Ki67 (MIB-1; Dako, Ely, UK), anti-p21 (C-19; Santa Cruz Biotechnology, Santa Cruz, CA) and anti-actin (Santa Cruz Biotechnology). The membranes were exposed to horseradish peroxidase (HRP)-conjugated secondary species-specific antibodies (Santa Cruz Biotechnology).

### Spherogenicity assay

Spherogenicity assays were performed by seeding the cells at 300 cells/well/100 µl in NB medium using 96-well plates and allowing them to form spheres for a period of at least 15 days. Spheres containing a minimum of six cells were scored. Data are expressed as mean ± SD normalized to control.

### Flow cytometry

The cells were dissociated with Accutase™ (Chemie Brunschwig, Basel, Switzerland). For cell cycle analysis 10^6^ cells/condition were fixed and permeabilized with cold 70% ethanol. After two PBS washes, the cells were treated with RNase A (Gibco, Grand Island, NY) for 30 min at 4 °C to remove RNA and ensure only DNA is stained with propidium iodide (PI) (Sigma-Aldrich). For cell death analysis, 10^5^ cells were re-suspended in Annexin buffer (10 mM HEPES, 140 mM NaCl, 2.5 mM CaCl_2_, pH = 7.4) and incubated with Annexin-V-FITC (BD Biosciences, Franklin Lakes, NJ) and PI (Sigma-Aldrich) containing 0.1% Triton X-100 (Sigma-Aldrich) for 15 min at room temperature. Cells were re-suspended in flow cytometry buffer (PBS, 0.5% BSA, 0.02% NaN3, 1 mM EDTA) and cell death and cell cycle phases were analyzed by flow cytometry in a BD FACSVerseTM flow cytometer (Becton Dickinson AG, Allschwil, Switzerland). Data were analyzed using FlowJo Software, version 10.0.8 (Ashland, OR).

### Enzyme-linked immunosorbent assays (ELISA)

Supernatants of 2 × 10^6^ cells were collected after 24 h and concentrated using an Amicon Ultra centrifugal filter (3 K) (Millipore, Temecula, CA). For quantitative detection of secreted HGF, a HGF ELISA kit (Invitrogen, Basel, Switzerland) was used.

### RNA interference

To silence the expression of *SMAD2*, *SMAD3*, *SMAD4*, or *ALK-5*, GIC were transiently transfected by electroporation (Neon transfection system, Invitrogen) using siRNA pools (ON-TARGET plus human SMAD2 siRNA-SMART pool L-003561-00, ON-TARGET plus human SMAD3 siRNA-SMART pool L-020067-00-0020, ON-TARGET plus human SMAD4 siRNA- SMART pool L-003902-00 and ON-TARGET plus human ALK-5 siRNA- SMART pool L-003929-00 (Dharmacon, Lafayette, CO). Non-targeting siRNA pool was used as a negative control. Lentiviral pGIPZ vectors encoding *c-MET-*specific (Oligo ID V3LHS_642486) or non-silencing control shRNA (Oligo ID RHS4346) were purchased from Thermo Scientific (Waltham, MA). Glioma cells were transduced with lentiviral particles produced as described^37^. Stably transduced clones were isolated with 4 µg/ml puromycin and subjected to analyses and assays after 1–5 passage post selection.

### Immunocytochemistry and immunohistochemistry

Glioblastoma patient specimens were obtained and analyzed in accordance with an Institutional Review Board-approved project plan (Kantonale Ethikkommission Zürich, Switzerland, KEK-ZH-Nr./BASCE-Nr. 2016-00456). Single- or double-antigen labeling of formalin-fixed and paraffin-embedded 4-μm-thick sections by immunocytochemistry (ICC) or immunohistochemistry (IHC) included the following steps: deparaffinization, rehydratation, boiling in EDTA buffer (1 mM EDTA, 0.05% Tween 20, pH 8.0) for 15 min, 1% H_2_O_2_ incubation for 15 min and blocking in SuperBlock solution (ScyTek Laboratories, Logan, UT) for 30 min, followed by primary antibody application. The following primary antibodies were used: rabbit anti-phospho-c-MET^Tyr1234/1235^ polyclonal (R&D Systems), rabbit IgG isotype control (Abcam), mouse monoclonal anti-TGF-β2 antibody (Abcam) or mouse IgG1 monoclonal (Abcam). Incubation with these was followed by HRP-conjugated anti-rabbit IgG secondary antibody (Santa Cruz) or the VECTASTAIN ABC-alkaline phosphatase (AP) detection system (containing biotinylated anti-mouse IgG) (Vector Labratories, Burlingame, CA). For color development Permanent HRP Green (Zytomed System, Berlin, Germany) and VectorRed (Vector Labratories) substrate kits were used.

Specificity verification and titration of primary and secondary antibodies were carried out first by ICC (Fig. S1). 3D-cultured GIC spheres were embedded into paraffin blocks according to a slightly modified protocol^38^, subsequently sectioned and subjected to standard staining procedures as specified above.

The evaluation of antigen expression was performed on tumor regions exhibiting histomorphological features of glioblastoma including the prominence of glomeruloid vessels, nuclear atypia, necrosis and high proliferative activity confirmed by Ki67 staining. The IHC-stained sections were viewed by a Nikon Eclipse 80i (Nikon Corporation, Tokyo, Japan) microscope with a Nikon CFI Plan Apo Lambda 40X (Nikon Corporation) bright field objective, Olympus UC30 (Olympus K.K., Tokyo, Japan) camera and processed by cellSens Entry 1.12 by Olympus Corporation (Olympus K.K.) software. To compare the proportion of each antigen to the other in the same tumor specimen, the quantification of each chromogen was performed using ImageJ (version 1.32j) software (National Institutes of Health, http://rsb.info.nih.gov/ij/). TGF-β2 and p-c-MET immunoreactivities were determined by measuring the integrated density (the sum of the pixel values) per field (tumor region). A cutoff of 5% stained tumor cells was used to allocate a sample to the TGF-β2 and p-c-MET high or low groups. The staining procedure, image acquisition, threshold settings were identical for the entire set of patient samples. Clinical information on the cohort of 58 newly diagnosed and 8 recurrent glioblastoma patients used in this study has been published^39^.

### Single-cell quantitative RT–PCR of reverse transcribed RNA

Surgically resected glioblastoma tissues were immediately digested with a papain-based dissociation system (Worthington, Lakewood, NJ). After digestion, leukocytes were depleted by using anti-human CD45-conjugated microbeads (Miltenyi Biotec, Bergisch Gladbach, Germany) and MACS LD columns (Miltenyi Biotec). The tumor cell population was filtered by excluding CD31-expressing cells. The single-cell quantitative RT–PCR (qRT–PCR) was performed at the Federal Institute of Technology (ETH) Zurich, Department of Biosystems Science and Engineering (D-BSSE) and Genomics Facility in Basel by using C1 Single-Cell Autoprep and BioMark HD instruments (Fludigim, San Francisco, CA). Cq values were converted to expression levels using the the equation Log_2_Ex = C_q LOD (Limit of Detection)_—Cq with a LOD C_q_ of 25 and data was mean-centered^40^.

### Statistical analysis

All in vitro experiments reported here were performed in biological and technical replicates. Quantitative data were expressed as the mean and SD of triplicate determinations. The statistical analyses were performed by Student’s *t*-test and one-way ANOVA with Tukey’s multiple comparison tests wherever applicable (GraphPad Software, La Jolla, CA). The Spearman’s rank correlation coefficient was calculated to analyze the statistical association between the mean of TGF-β2 and p-c-MET levels (integrated density values) in vivo.

## Results

### TGF-β suppresses HGF/c-MET pathway activity in glioblastoma

**Fig. 1:**
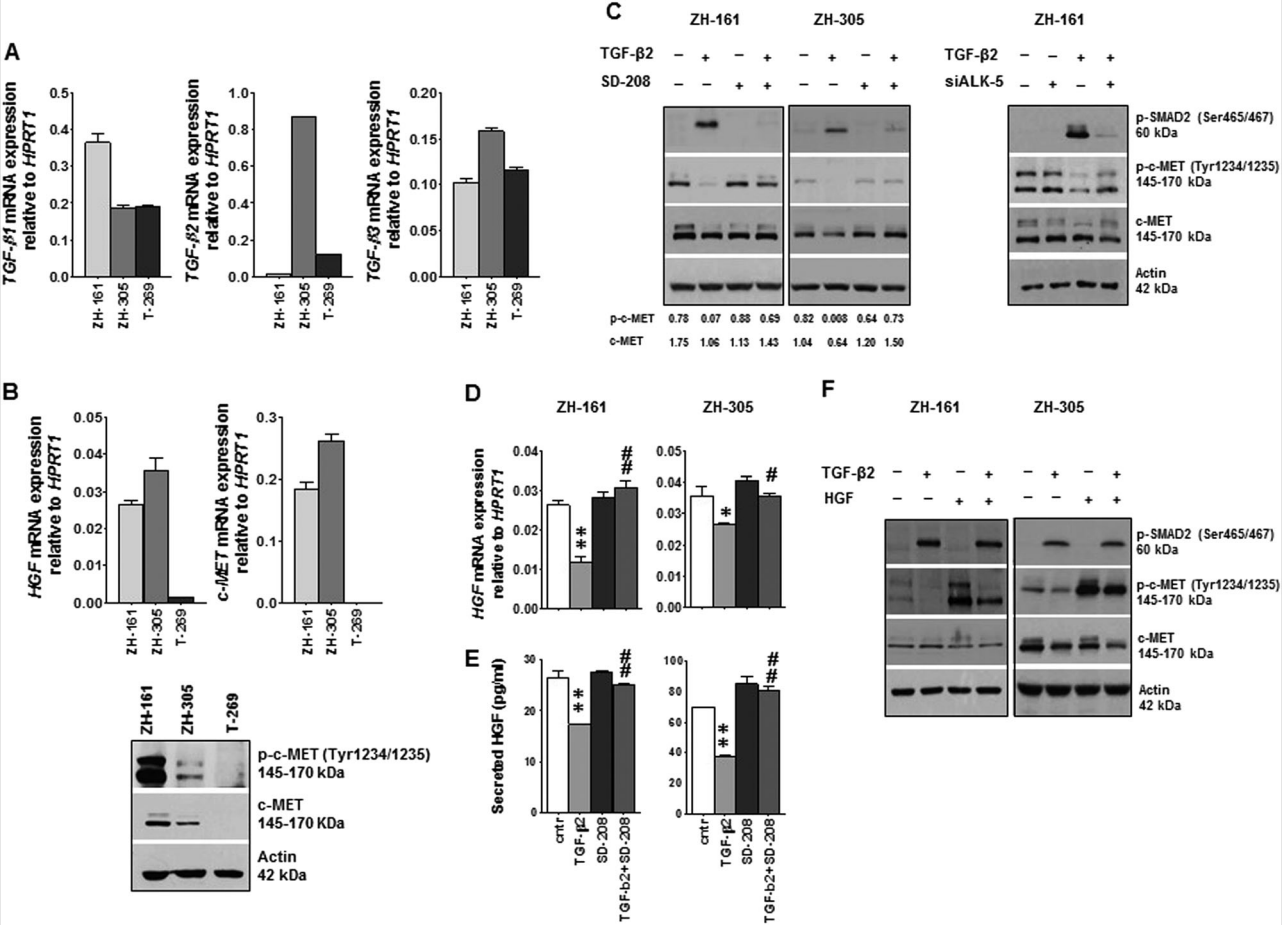
Control of c-MET activity by TGF-β signaling **a**, **b** GIC were assessed for *TGF-β 1/2/3* (**a**) and *HGF*, *c-MET* (**b**) mRNA levels by RT–PCR. Equal amounts of cellular lysates were assessed also for c-MET and p-c-MET levels by immunoblot (**b**). **c** GIC were seeded in complete NB medium in the presence of SD-208 (1 µM) or TGF-β2 (2 ng/ml) or both, or ALK-5 siRNA (100 nM) with or without TGFβ2 (2 ng/ml) for 24 h. DMSO diluted in NB medium (1:20’000) served as a control. p-SMAD2, p-c-MET and c-MET protein levels were assessed by immunoblot; 30 µg/lane protein for ZH-161 and 50 µg/lane for ZH-305 were loaded. Actin was used as loading control. Quantification of band intensity by ImageJ is shown below the immunoblot panels. **d**, **e** Modulation of *HGF* mRNA expression (**d**) and HGF protein release into the supernatant (**e**) by TGF-β2 stimulation in the absence or presence of SD-208 was assessed by RT–PCR and by ELISA (*p < 0.05, **p < 0.01, effect of TGF-β2 compared to control, ^#^ *p < *0.05, ^##^ *p < *0.01 effect of TGF-β2 and SD-208 co-treatment compared to TGF-β2 alone). **f** The interference of TGF-β2 (2 ng/ml) with HGF (50 ng/ml) stimulation of c-MET phosphorylation was assessed by immunoblot

Patient-derived GIC models referred to as ZH-161, ZH-305 and T-269 were first analyzed for their TGF-β and HGF/c-MET status to characterize the baseline actvity of both pathways. All three cell lines expressed *TGF-β1* and *TGF-β3* mRNA. TGF-β2 was predominant in ZH-305 whereas ZH-161 was *TGF-β2*-negative at mRNA and protein level. (Fig. 1a and Supplementary Fig. S1A, B). Consistent with the expression of *HGF* and *c-MET* mRNA, ZH-161 and ZH-305 exhibited basal levels of phosphorylated c-MET (p-c-MET) as demonstrated by immunoblot and immunocytochemistry (Fig. 1b and Supplementary Fig. S1A, B). The expression of *HGF* and *c-MET* mRNA as well as the levels of c-MET and p-c-MET protein were below detection limit in T-269 (Fig. 1b). Therefore, T-269 was used as a negative, whereas ZH-161 and ZH-305 were used as positive models for c-MET-dependent GIC. Despite expression of various *TGF-β* family members, cellular p-SMAD2 levels were low or undetectable (Fig. 1c). First, we monitored whether TGF-β stimulation modulates baseline c-MET activity using c-MET-positive GIC models. Exposure of ZH-161 or ZH-305 cells to recombinant TGF-β1-induced or TGF-β2-induced activation of TGF-β signaling, as defined by increased p-SMAD2 levels, and resulted in significant reduction of p-c-MET (Fig. 1c and Supplementary Fig. S2A, B). A minor decrease of the total c-MET was observed upon TGF-β1 and TGF-β2 stimulation, too. Two different TGF-βRI (ALK-5) inhibitors, SD-208 and LY2157299, did not affect basal p-c-MET levels, but prevented the loss of p-c-MET in response to exogenous TGF-β2 (Fig. 1c and Supplementary Fig. S2C). Consistent with the pharmacologic inhibition of ALK-5, its genetic silencing rescued p-c-MET levels in TGF-β2-treated cells (Fig. 1c and Supplementary Fig. S2D). The negative regulation of p-c-MET detected by immunoblot was demonstrated by ICC (Supplementary Fig. S2E). The downregulation of p-c-MET was paralleled by a suppression of HGF mRNA expression and protein release and this effect was prevented by SD-208 in both cell lines (Fig. 1d, e). In contrast, *c-MET* mRNA expression was down-regulated by exogenous TGF-β2 in ZH-305 only and remained stable with co-treatment of TGF-β2 and SD-208 in both cell lines (Supplementary Fig. S2F). Furthermore, exogenous HGF rescued p-c-MET levels in TGF-β-treated cells (Fig. 1f), suggesting loss of *HGF* expression as a mechanism of negative regulation of the c-MET pathway by TGF-β.

### TGF-β activity on HGF/c-MET is regulated by MAPK/**ERK** and AKT signaling

To clarify the downstream signaling events resulting in suppression of c-MET pathway activity, we first analyzed the involvement of SMAD proteins, the central effectors of TGF-β canonical signaling. We used siRNA oligonucleotides to specifically silence the expression of *SMAD2*, *SMAD3* or *SMAD4* (Supplementary Fig. S3A–C). Similarly to the *ALK-5, SMAD2/3/4* gene silencing did not alter gene expression of the major effector and downstream target of TGF-β, *PAI-1* (Supplementary Figs. S2D and S3D). Exogenous TGF-β2-dependent upregulation of *PAI-1* was lost in *SMAD* knockdown cells, proving the efficiency of *SMAD* silencing (Supplementary Fig. S3D).

In contrast to TGF-βRI inhibition, *SMAD* silencing did not counteract the TGF-β2-evoked suppression of p-c-MET. However, *SMAD4* siRNA increased p-c-MET in both GIC lines in the absence of exogenous TGF-β2 (Supplementary Fig. S3B). A similar effect was also observed with *SMAD2* silencing in ZH-305 cells (Supplementary Fig. S3C).

**Fig. 2:**
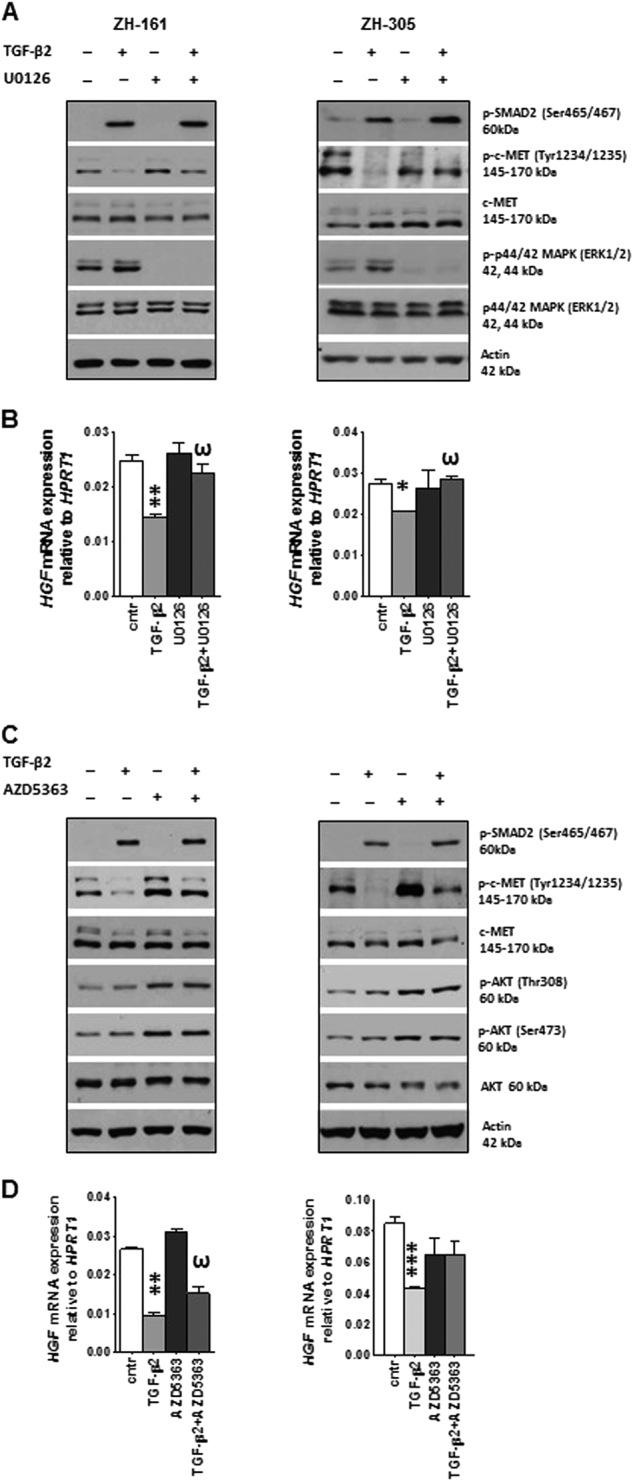
Analysis of TGF-β downstream signaling components on c-MET activity **a** GIC were treated with U0126 (10 µM) or TGF-β2 (2 ng/ml) alone or combination for 16 h, and assayed for modulation of c-MET activity by immunoblot. **b** Modulation of *HGF* mRNA by the same treatment (4 h) in both cell lines was evaluated by RT–PCR. **c** Quantification of c-MET activity and** d** . *HGF* mRNA levels upon AZD5363 (3 µM) or TGF-β2 (2 ng/ml) treatment or their combination (4 h) was performed by immunoblotting and RT–PCR, respectively. TGF-β2 was added 1 h later than U0126 and AZD5363. DMSO diluted in NB medium (1:3’300) was used as a control (*p < 0.05, **p < 0.01, effect of TGF-β2 compared to control, ^ω^ *p* < 0.05, effect of TGF-β2 and U0126 or TGF-β2 and AZD5363 co-treatment compared to TGF-β2 alone)

Since TGF-β2 induces ERK phosphorylation in a U0126-sensitive manner in glioma cells (Fig. 2a and Supplementary Fig. S4A), we next explored a role for ERK in controlling the HGF/c-MET pathway. U0126 alone altered neither c-MET phosphorylation nor *HGF* mRNA levels but counteracted the inhibitory effect of exogenous TGF-β2 on p-c-MET and *HGF* mRNA in ZH-161 and ZH-305 (Fig. 2a, b). Moreover, U0126 prevented the downregulation of *c-MET* mRNA expression by TGF-β2 in ZH-305 (Supplementary Fig. S4B). The involvement of PI3K/AKT signaling in TGF-β-dependent control of c-MET pathway was also addressed using the AKT inhibitor, AZD5363. Exposure to TGF-β2 did not modulate p-AKT^Thr308/Ser473^ and AZD5363 alone did not modify *HGF* expression. However, the inhibition of p-AKT by AZD5363 induced c-MET phosphorylation and the levels of p-c-MET remained unchanged when the cells were treated with TGF-β in the presence of AZD5363 (Fig. 2c). Furthermore, the downregulation of *HGF* or *c-MET* mRNA expression by TGF-β2 was attenuated or prevented by AZD5363 (Fig. 2d and Supplementary Fig. S4C). The increase of *c-MET* mRNA by AZD5363 did not translate into protein level (Fig. 2c and Supplementary Fig. S4C). The increase of p-AKT (Ser473, Thr308) in both ZH-161 and ZH-305 (Fig. 2c) reflects the mechanism of drug action^41^.

### Context-dependent role of HGF/c-MET and TGF-β signaling in stemness

**Fig. 3:**
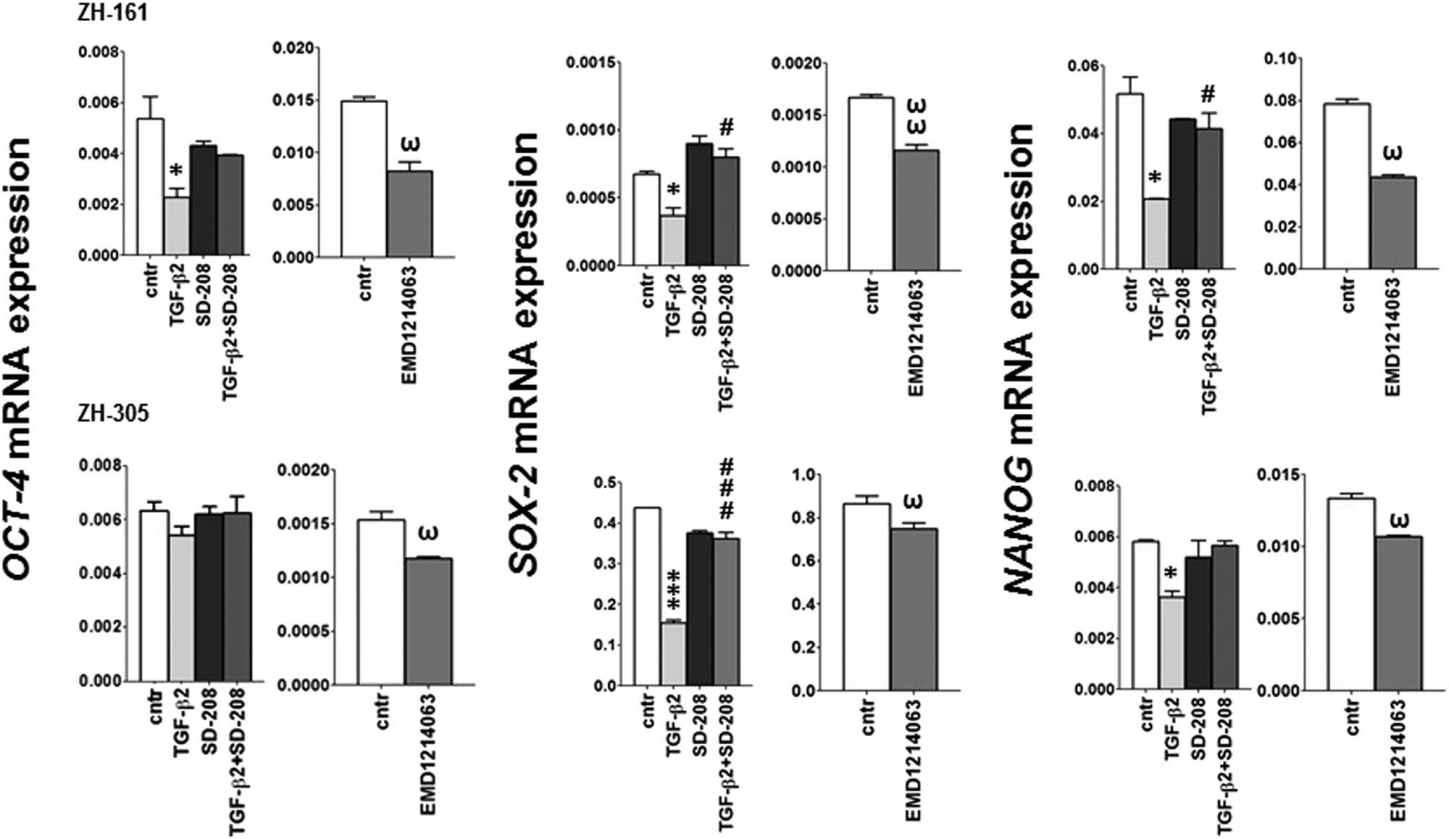
Modulation of stem cell marker expression by TGF-β or c-MET modulation *OCT-4*, *SOX-2* or *NANOG* gene expression upon exposure to SD-208 (1 µM), TGF-β2 (2 ng/ml) or both (4 h) were assessed by RT–PCR (*p < 0.05, ***p < 0.001, effect of TGF-β2 relative to control; ^#^ *p < *0.05, ^###^ *p < *0.001 effect of TGF-β2 and SD-208 relative to TGF-β2). The c-MET inhibitor EMD1214063 (200 nM) was included as a reference (^ω^ *p *< 0.05, ^ωω^ *p* < 0.01)

We next sought to investigate the functional relevance of the interactions observed between the TGF-β and c-MET pathways. Given their contribution to the maintenance of stem-like features of GIC, we first evaluated how these two pathways alone and their balance influence the expression of stem-like markers and self-renewal capacity. We treated the cells with TGF-β2, or the ALK-5 inhibitor, SD-208, or the c-MET inhibitor, EMD1214063. SD-208 alone did not affect OCT-4 (octamer transcription factor), SOX-2 (SRY (sex determining region Y)-box 2) or NANOG (Nanog homeobox) mRNA expression. Approximately 2-fold decreases of these SC marker mRNA expression levels were observed upon TGF-β2 stimulation in ZH-161 and ZH-305 cells, except OCT-4 in ZH-305. This reduction was prevented by SD-208. Similar to TGF-β2, inhibition of c-MET by EMD1214063 resulted in transcriptional repression of these genes (Fig. 3). In contrast, none of these markers were affected by TGF-β2 or EMD1214063 in HGF/c-MET-negative T-269 cells (Supplementary Fig. S5A).

**Fig. 4:**
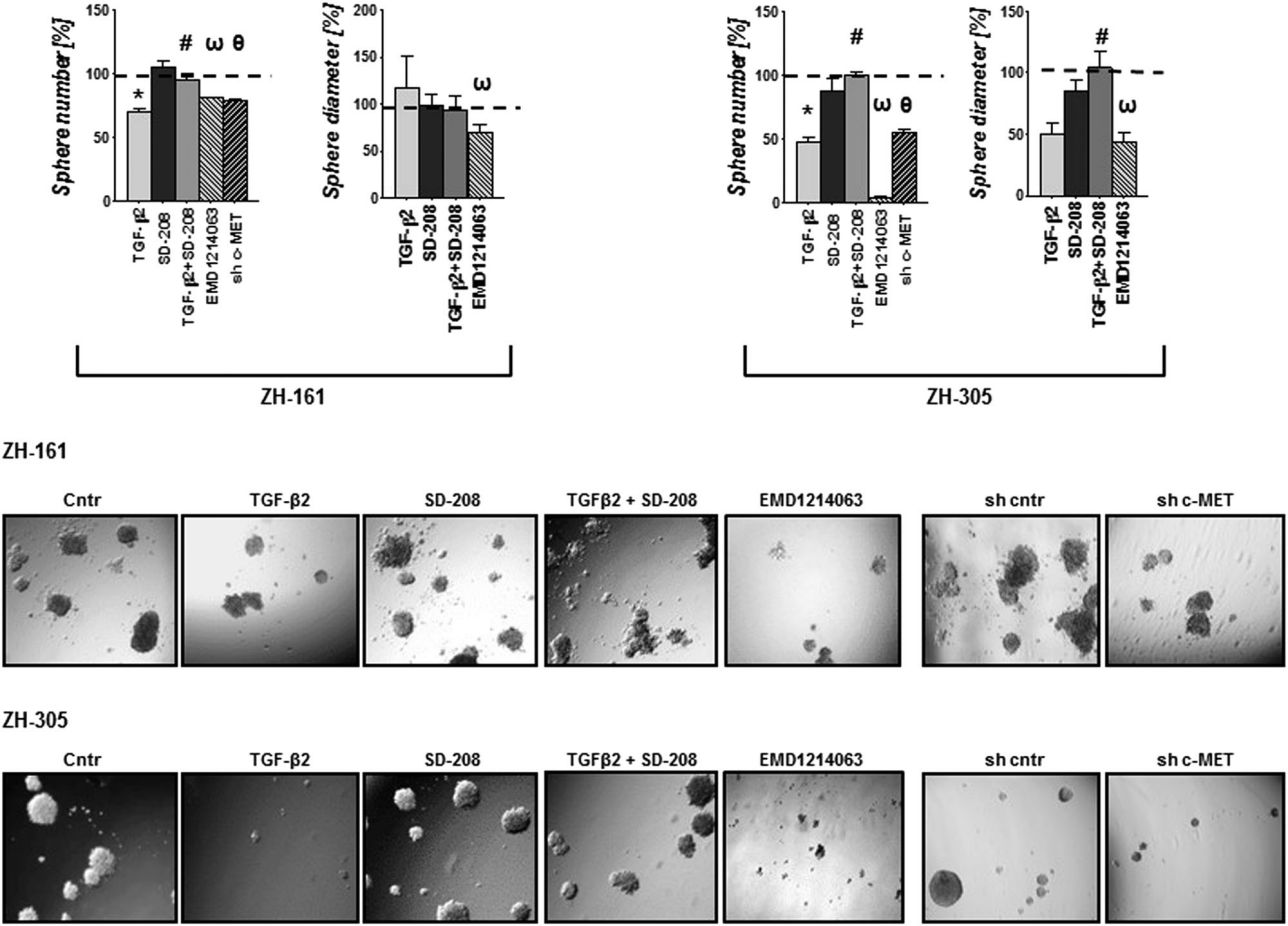
Control of spherogenicity by TGF-β or c-MET pathway ZH-161 or ZH-305 cells were plated at 300 cells/well in NB medium in the absence or presence of SD-208 (1 µM) or TGF-β2 (10 ng/ml) or SD-208 combined with TGF-β2 in 96-well plates. *c-MET* shRNA expressing or EMD1214063 (200 nM)-treated ZH-161 or ZH-305. Sphere formation was assessed at day 21 in triplicates (*p < 0.05, effects of TGF-β2 compared to control, ^#^ *p < *0.05, effect of TGF-β2 and SD-208 co-treatment compared to TGF-β2 alone; ^ω^ *p* < 0.05, effect of EMD1214063 compared to control, ^θ^ *p < *0.05, effect of c-MET shRNA compared to control). Spherogenicity was calculated based on the number of spheres and average size of the spheres as indicated (upper panels). Representative photomicrographs showing sphere formation in each condition for ZH-161 (middle panels) and ZH-305 (lower panels). The photomicrographs were taken using a 5X objective

Accordingly, TGF-β2-treated ZH-161 or ZH-305 showed decreased spherogenesis in a SD-208-sensitive manner. Similarly, lentivirus-mediated c-MET gene silencing or c-MET inhibition with EMD1214063 reduced spherogenicity, but none of that was seen in T-269 cells (Fig. 4, Supplementary Fig. S4D showing the knockdown efficiency and Supplementary Fig. S5B). The strong effects on spherogenicity in ZH-161 and ZH-305 may involve minor cytotoxic and antiproliferative effects of TGF-β2 or EMD1214063 as demonstrated by changes in viability, cell cycle progression and increase of the cyclin-dependent kinase inhibitor p21 under these conditions (Supplementary Fig. S6A–C). To evaluate whether the TGF-β2 effect on stemness was due to its negative control of HGF/c-MET, we analyzed the spherogenicity upon TGF-β2 exposures in the presence of recombinant HGF. Interestingly, exogenous HGF did not promote spherogenicity under these culture conditions, however, profound induction of sphere formation was seen when EGF and FGF were omitted from the medium in ZH-161 and ZH-305 except T-269. In addition, the stimulation with HGF overcame the reduction in sphere formation observed upon TGF-β2 in ZH-161 and ZH-305 (Supplementary Fig. S7).

### Detection and significance of intratumoral TGF-β and HGF/c-MET heterogeneity in glioblastoma

The findings of an inhibitory effect on HGF/c-MET by TGF-β in vitro prompted us to validate an association between the two pathways in vivo using human glioblastoma specimens. The specificity of the antibodies and the optimal conditions for IHC were determined using paraffin-embedded positive and negative control cell lines. *TGF-β2* mRNA-expressing ZH-305 cells were immunopositive whereas *TGF-β2* mRNA-negative ZH-161 cells were also negative by ICC (Fig. 1a and Supplementary Fig. S1A). Similar studies were performed to test the anti-phospho-c-MET antibody in ZH-161 and T-269 cells which were also characterized for their p-c-MET status by immunoblot (Fig. 1b and Supplementary Fig. S1B).

**Fig. 5:**
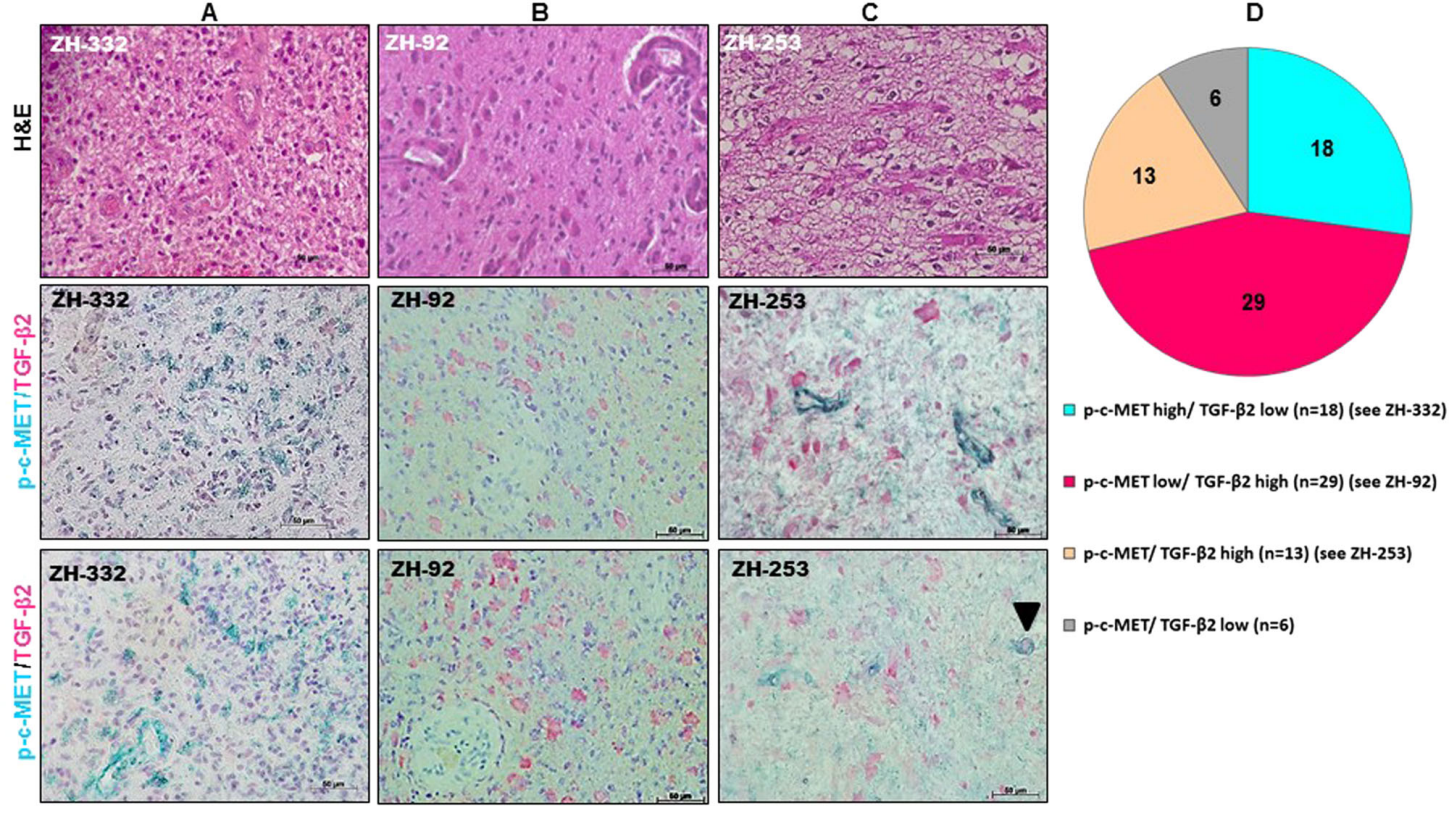
Simultaneous detection of TGF-β2 and phosphorylated c-MET in glioblastoma Double immunostaining was performed on formalin-fixed, paraffin-embedded surgical glioblastoma specimens. The quantification of each chromogen was performed separately within three randomly selected different microscopic fields of each specimen. Blood vessels were excluded from the measurement. **a**–**c** Representative images show hematoxylin and eosin (H&E) staining (upper panels); p-c-MET immunoreactivity in green ((**a**, **c**) middle and lower panels) and TGF-β2 in red ((**b**, **c**) middle and lower panels) in tumor regions of three patients. Nuclei are counter-stained with hematoxylin (blue). The scale bars correspond to 50 μm. **d** Graphical representation of the percentage of glioblastomas high/low for either TGF-β2 or p-c-MET or both

We co-stained glioblastoma tissue samples from a large cohort of 66 different glioblastoma patient for TGF-β2 (red) and p-c-MET (green) (Fig. 5a–c). p-c-MET was detected in glioblastoma cells as well as in the vasculature. Double staining of TGF-β2 and p-c-MET allowed the classification of the tumor specimens into four groups (Fig. 5d). In line with our in vitro findings of a negative control by TGF-β of c-MET activation, the two antigens were rarely present simultaneously in the same tumor specimen (*n* = 13). Moreover, co-expression of TGF-β2 and p-c-MET in the same tumor cells was rarely detected (Fig. 5c, see arrowhead), showing that these two antigens exhibit almost mutually exclusive staining patterns among patients and within the tumor from the same patient. This was reflected in a negative Spearman correlation between TGF-β2 and p-c-MET levels (*r* = −0.34, *p* = 0.005).

### Correlations among *TGF-β*, *HGF* and stem cell markers in single-cell qRT–PCR analysis using freshly resected glioblastoma tissues

**Fig. 6:**
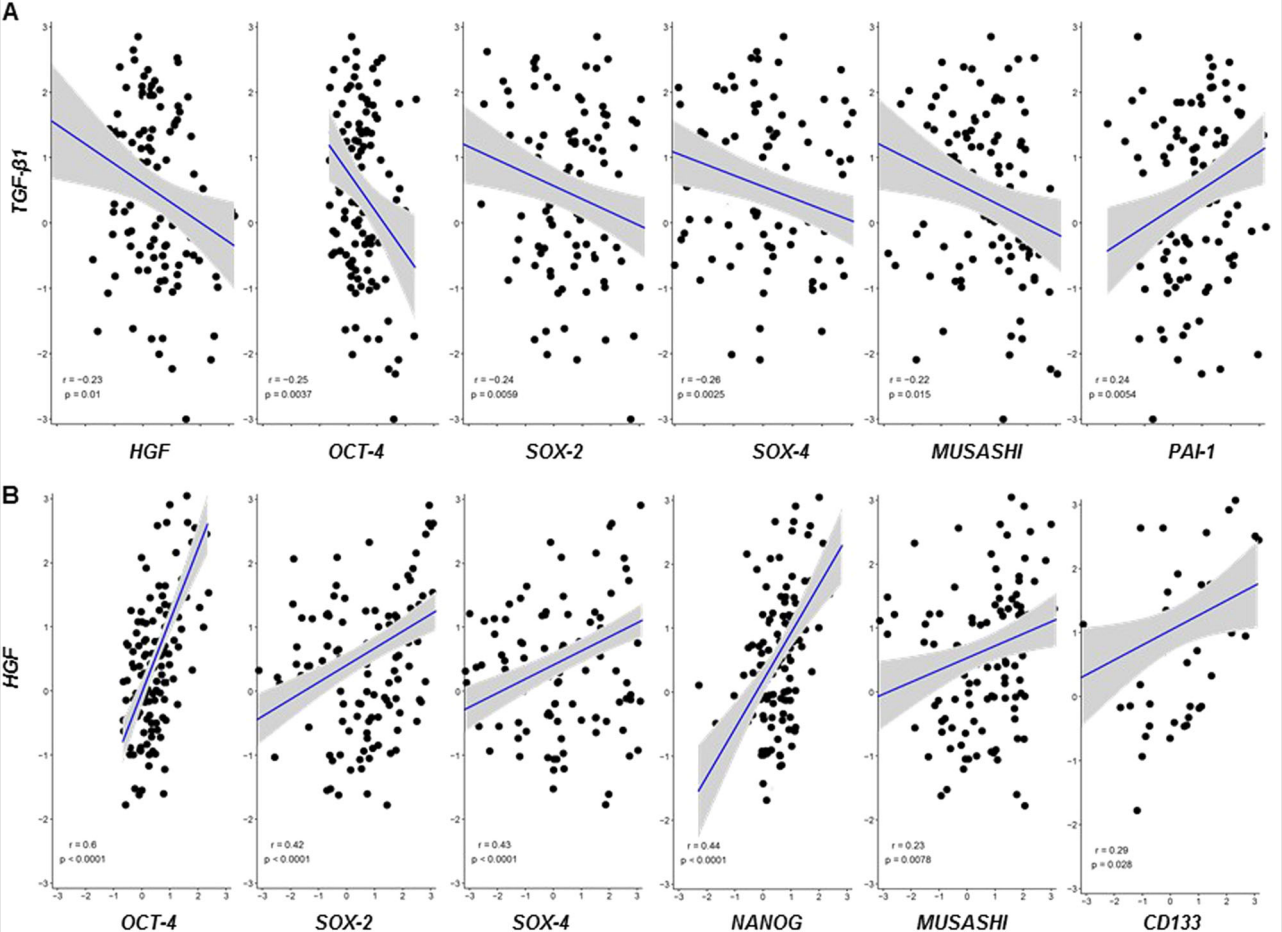
Correlations among *TGF-β*, *HGF* and stem cell markers upon single-cell qRT–PCR analysis using freshly resected glioblastoma tissues **a**, **b** Pairwise complete correlations of *TGF-β1* vs. *HGF*, *OCT-4*, *SOX-2*, *SOX-4*, *MUSASHI*, *PAI-1* (*a*, and *HGF* vs. *OCT-4*, *SOX-2*, *SOX-4*, *NANOG*, *MUSASHI*, and *CD133* **(b**) mean-centered LOG_2_Ex values for 437 individual glioblastoma cells from 6 patients are shown. Pearson’s correlation coefficients are depicted after Bonferroni correction for multiple testing. Pearson’s correlation coefficients for pairwise complete observations were computed using the corrplot R package version 0.77 and Bonferroni correction was applied to adjust for multiple hypothesis testing. Scatterplots of significant correlations were plotted using the ggpubr R package version 0.1.2. Extreme C_q_ values of “999” were excluded and low quality cells that did not express the housekeeping genes *ARF-1* were discarded. The primer sequences are provided with Supplementary Table S1

Finally, we expanded our study to analyze expression profiles of *TGF-β*, *HGF*, and a panel of genes essential for pluripotency in the CD45^-^/CD31^-^ presumptive tumor cell population isolated from freshly resected tumor tissue of 6 glioblastoma patients at diagnosis. Single-cell gene expression analysis revealed that *TGF-β1* gene demonstrated the highest and *TGF-β3* the lowest expression level of the three family members. Only *TGF-β1* levels correlated positively with TGF-β target gene, *PAI-1* (Fig. 6a). *HGF* gene expression positively correlated with several SC markers (Fig. 6b). Importantly, there were negative correlations between *TGF-β1* and *HGF* expression as well as *TGF-β1* and a panel of SC markers (Fig. 6a). No significant relationship between *TGF-β 2/3* and HGF or SC factors was identified.

## Discussion

Glioblastoma is a universally fatal type of cancer characterized by molecular diversity and intratumoral heterogeneity^42^. Potentially as a consequence of the heterogeneity of functionally redundant cell signaling networks and multiple genetic alterations^43^, glioblastoma remains largely refractory to current approaches of cancer therapy targeting specific signaling pathways^44^. Given their role in mediating migration and invasiveness, resistance to irradiation and maintenance of the glioma SC pool, both HGF/c-MET and TGF-β signaling pathways have been considered as potential therapeutic targets. Several approaches evaluated the inhibition of TGF-β signaling in glioma (www.clinicaltrials.gov) using antibodies, antisense oligonucleotides, ligand traps or TGF-β receptor kinase inhibitors^29,45^. Similarly, many clinical trials explored a therapeutic role for HGF/c-MET inhibition, but none of these approaches have been successful^30,31,46^. The TGF-β pathway suppresses mammary tumorigenesis by antagonizing HGF/c-MET signaling in fibroblasts^47,48^. Moreover, TGF-β negatively regulates *c-MET* and *HGF* mRNA levels in human squamous carcinoma cells^49^. In astrocytoma cells, TGF-β family ligands decrease HGF synthesis and secretion^50^. However, the functional interactions between TGF-β family members and HGF/c-MET signaling in glioblastoma remain uncharacterized.

Here we have elucidated the complex crosstalk between the TGF-β- and HGF-dependent signaling pathways in gliobastoma using three patient-derived GIC models as well as human glioblastoma specimens. We selected ZH-161 and ZH-305 models with constitutive c-MET activation and included a c-MET-negative model, T-269, as a reference (Fig. 1b). We found that TGF-β stimulation reduced phospho-c-MET in ZH-161 and ZH-305 cells (Fig. 1c). This was explained by a reduction of HGF mRNA expression and protein release (Fig. 1d, e). Exogenous HGF rescued c-MET pathway activity in the presence of TGF-β (Fig. 1f). In addition, repression of p-c-MET was associated with minor reduction of c-MET at mRNA and protein levels in ZH-305 cells (Fig. 1c and Supplementary Fig. S2F).

The effect of TGF-β on HGF/c-MET pathway activation was prevented upon pharmacological or genetic inhibition of ALK-5 (Fig. 1c, Supplementary Fig. S2C,D). Silencing of *SMAD2* or *SMAD4* gene expression increased basal p-c-MET levels, indicating a negative regulation of c-MET by SMAD at basal levels (Supplementary Fig. S3). In contrast with *SMAD2/3/4*, only the blockade of the MAPK/ERK cascade by U0126 and the PI3K/AKT signaling by AZD5363 attenuated the effect of TGF-β on c-MET phosphorylation as well as *HGF* gene expression (Fig. 2). TGF-β leads to the phosphorylation of ERK 1/2 (Supplementary Fig. S4A—2 h TGF-β—stimulation), thus defining this pathway probably as the major mediator of the crosstalk between TGF-β and c-MET.

In response to exogenous TGF-β, there was only minor or no stimulation of p-AKT (Fig. 2c), indicating a lack of direct interaction of TGF-β and PI3K/AKT in these GIC lines. Therefore, AKT acts as an independent mechanism to modulate the effects of TGF-β on c-MET. Interestingly, induction of c-MET phosphorylation was observed upon the blockade of PI3K/AKT signaling, confirming a negative control of c-MET activity by AKT and warranting clinical investigation to determine the significance of p-c-MET as an escape mechanism to AKT inhibitors (Fig. 2).

The PI3K/AKT and ERK signaling cascades are activated in response to c-MET and are common downstream effectors of many receptor tyrosine kinases^19,51^. Some evidence supports that PI3K/AKT and ERK act downstream of TGF-β, too^22,52^.

The low baseline TGF-β pathway activity in our GIC models is reflected by undetectable phospho-SMAD2/SMAD3 by immunoblot (Fig. 1c), furthermore by the unaltered gene expression of the major downstream effector gene of TGF-β, *PAI-1* upon *ALK-5* or *SMAD2/3/4* gene silencing (Supplementary Figs. S2D and S3D). Thus, reduced TGF-β shifts the balance in favor of c-MET receptor activation in these GIC lines. Notably, *PAI-1* transcriptional regulation is known to be stimulated by a variety of cytokines, including EGF^53,54^ independenly of TGF-β, which can explain the relative high basal *PAI-1* mRNA levels in these cells.

**Fig. 7:**
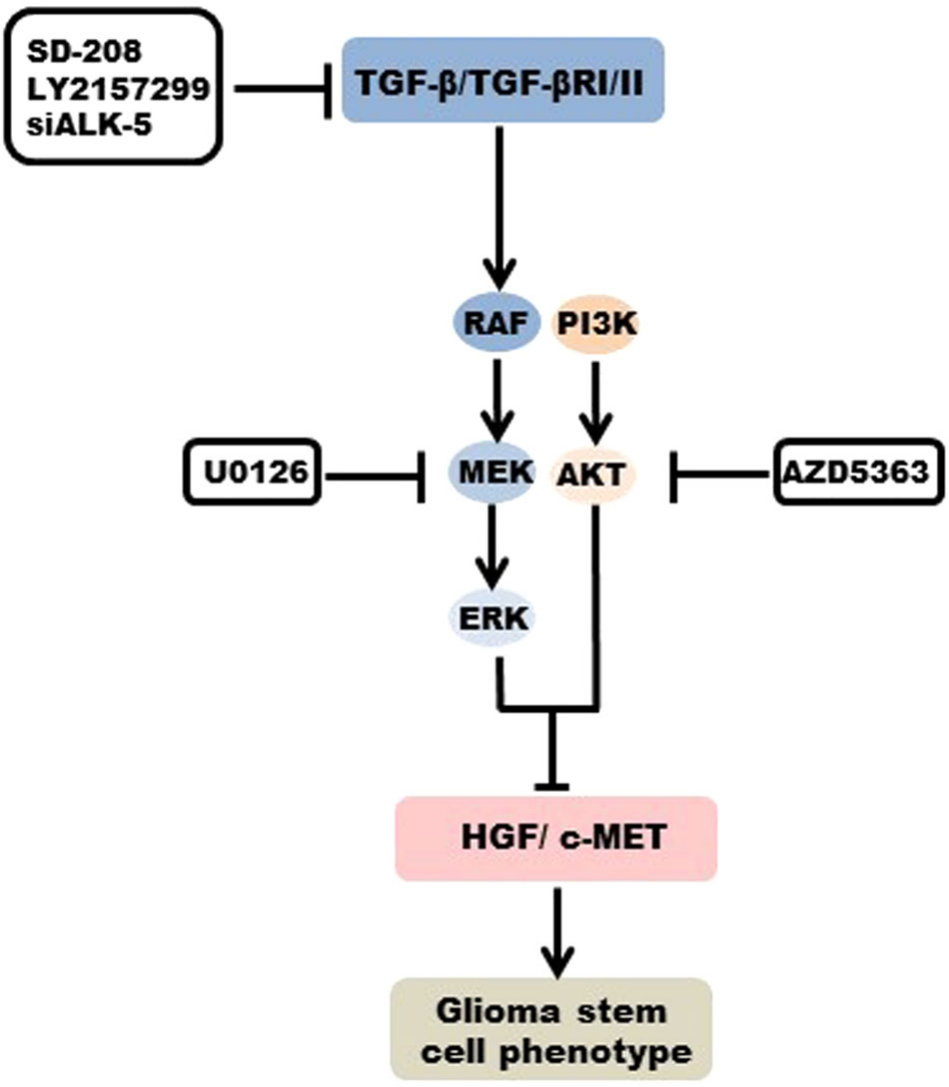
Overview of the control of the HGF/c-MET pathway by TGF-β in glioblastoma Illustration of the regulatory mechanisms involved in the control of c-MET activity by TGF-β, which determine the stem cell phenotype in c-MET-positive human glioma cells

The TGF-β and HGF/c-MET pathways have been attributed a role in induction of stem marker factors essential for GIC maintenance^9–11,55,56^. OCT-4, NANOG and SOX-2 contribute to the hallmark characteristics of stem and putative cancer stem cells by activation of target genes that encode pluripotency and self-renewal mechanisms^57^. We observed that exposure to TGF-β downregulated SC marker gene expression in c-MET-positive ZH-161 and ZH-305, but not in c-MET-negative T-269 cells (Fig.3 and Supplementary Fig. S5A). This was paralleled by an according suppression of spherogenicity at 21 days and minor changes in viability and cell cycle progression at 72 h (Fig. 4 and Supplementary Fig. S6). c-MET inhibition had the same effects as TGF-β stimulation in these assays. Conversely, T-269 did not respond to any manipulation of the TGF-β or c-MET pathways (Supplementary Fig. S5B), showing a paradigm of how SC behavior is differentially regulated in different cellular contexts. As previously observed^11,23,26,58^, we induced sphere formation by stimulation of c-MET positive GIC models with HGF only in the absence of EGF and FGF supplementation (Supplementary Fig. S7). In addition, under these culture conditions, the stimulation with HGF overcame the reduction in sphere formation observed upon exposure to TGF-β2 in ZH-161 and ZH-305 (Supplementary Fig. S7). These findings suggest that TGF-β may suppress SC characteristics by preventing c-MET overactivation in subgroups of gliomas (Fig. 7).

Taken together, the observations allowed to predict that human glioblastomas would tend to up-regulate either of both pathways, but not both signaling simultaneously. This assumption was confirmed by a negative correlation between TGF-β2 and p-c-MET immunoreactivity upon double immunostaining in a large cohort of glioblastoma patients (Fig. 5) as well as by a negative correlation between *TGF-β1* and *HGF* gene expression at single-cell level in freshly resected glioblastoma tissues (Fig. 6a).

The positive correlation between *HGF* and SC marker gene expression detected by single-cell qRT–PCR analysis, supports the notion that the SC population is enriched in HGF/c-MET-positive cells (Fig. 6b). Conversely, *TGF-β1* mRNA level negatively correlated with the gene expression of *HGF* as well as a panel of genes related to stemness. *TGF-β1/2/3* mRNA levels did not correlate in single-cell analysis (data not shown). *TGF-β1/2/3* mRNA levels do not allow the prediction of the respective protein levels as it was previously shown^39^. In vitro, TGF-β1 and TGF-β2 proteins were equally effective in controlling HGF/c-MET signaling and self-renewal (Fig. 1c; Supplementary Figs. S2A and S5C). Nevertheless, this data set strongly corroborates the newly identified suppressive role of TGF-β in HGF/c-MET-positive stem-like cells as shown in our preclinical and clinical analyses.

The modulation of c-MET pathway activity by TGF-β allows to speculate that subsets of patients with glioblastoma would have responded to TGF-β inhibitors such as galunisertib (LY2157299 monohydrate) with an increase of c-MET activity and potentially MET-mediated migration and invasion. This might have counteracted any potential benefit from TGF-β pathway inhibition^2^. Accordingly, c-MET negativity could be explored as a predictive biomarker for future clinical trials exploring TGF-β inhibition in glioblastoma.

## Electronic supplementary material

Figure S1

Figure S2

Figure S3

Figure S4

Figure S5

Figure S6A,B

Figure S6C

Figure S7

Supplementary materials
