# Supplementary figures and images for: Negative control of the HGF/c-MET pathway by TGF-β: a new look at the regulation of stemness in glioblastoma
Source: Cell Death Dis. 2017 Dec 13;8(12):3210. doi: 10.1038/s41419-017-0051-2 (PMC5870582; doi:10.1038/s41419-017-0051-2)

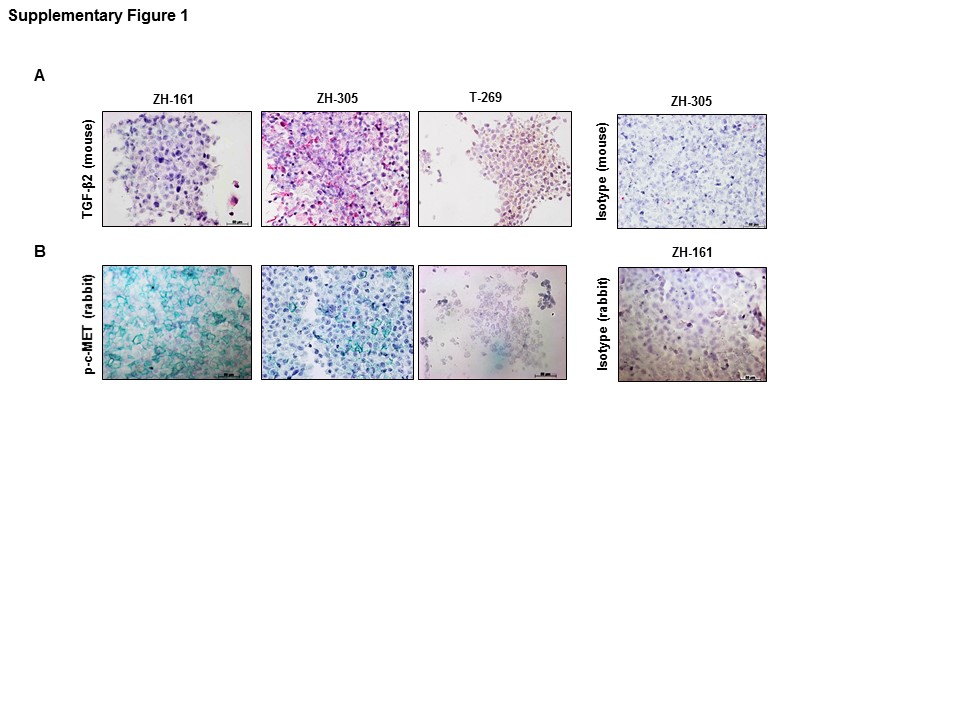

Supplement: Supplementary file 1 — Figure S1 [file 41419_2017_51_MOESM1_ESM.jpg]

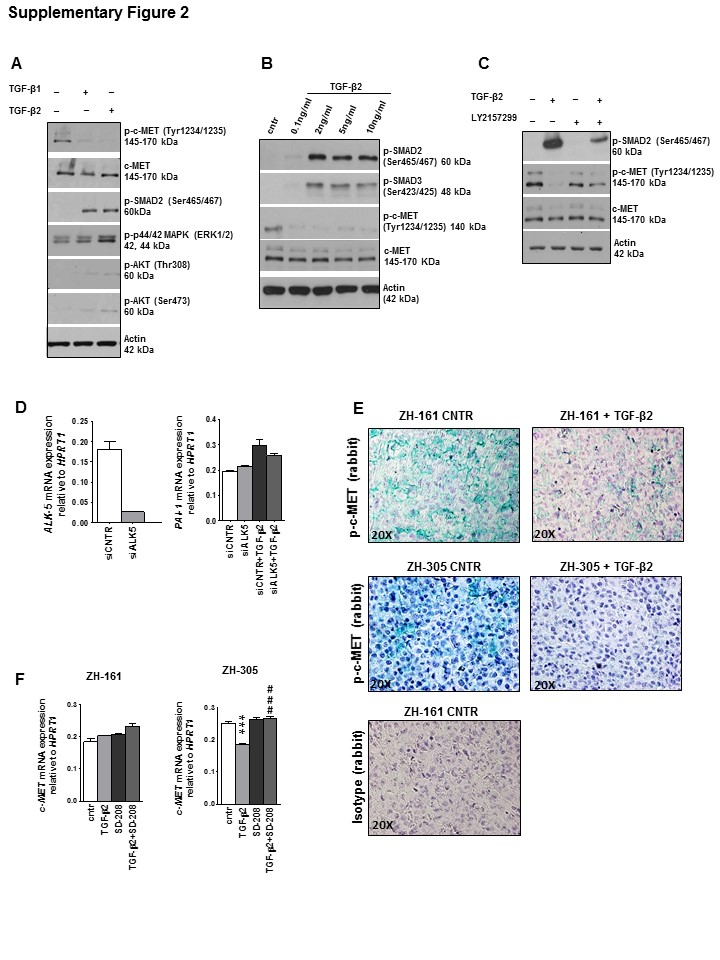

Supplement: Supplementary file 2 — Figure S2 [file 41419_2017_51_MOESM2_ESM.jpg]

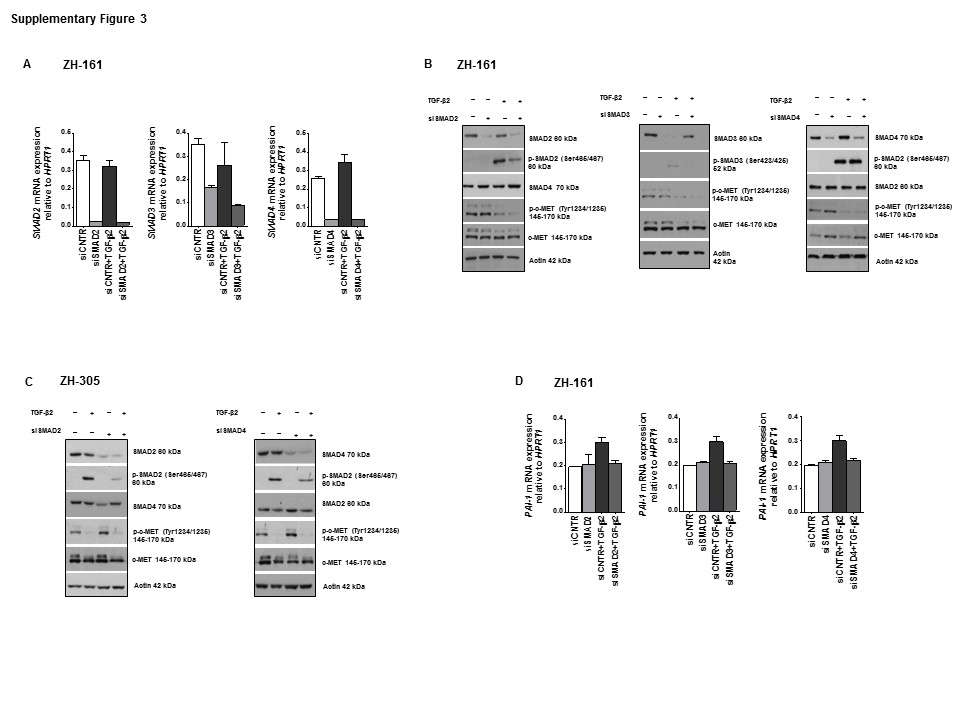

Supplement: Supplementary file 3 — Figure S3 [file 41419_2017_51_MOESM3_ESM.jpg]

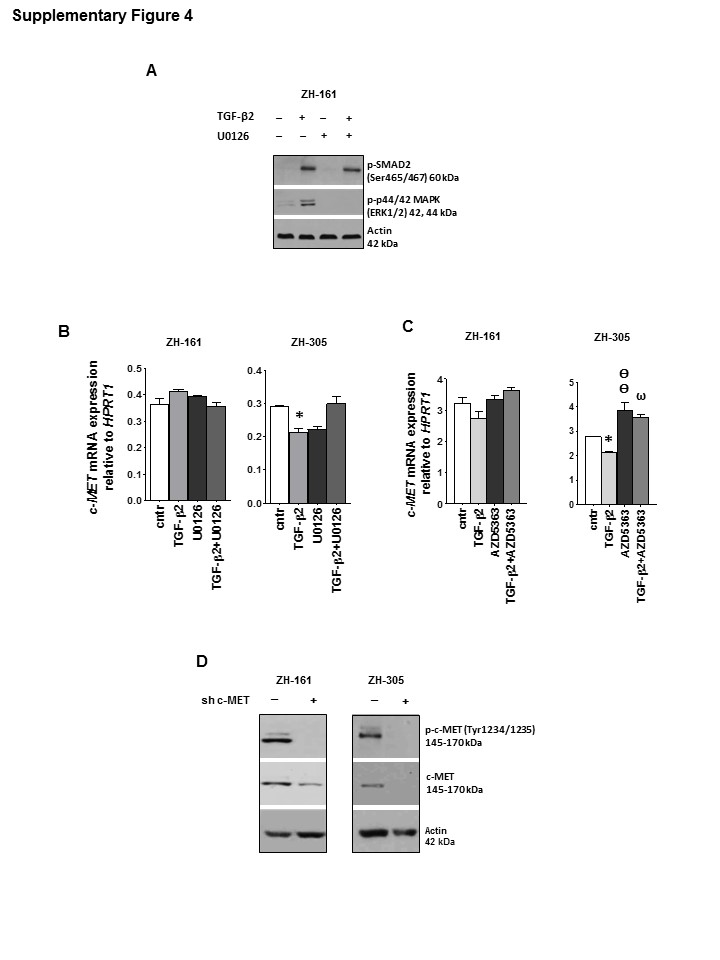

Supplement: Supplementary file 4 — Figure S4 [file 41419_2017_51_MOESM4_ESM.jpg]

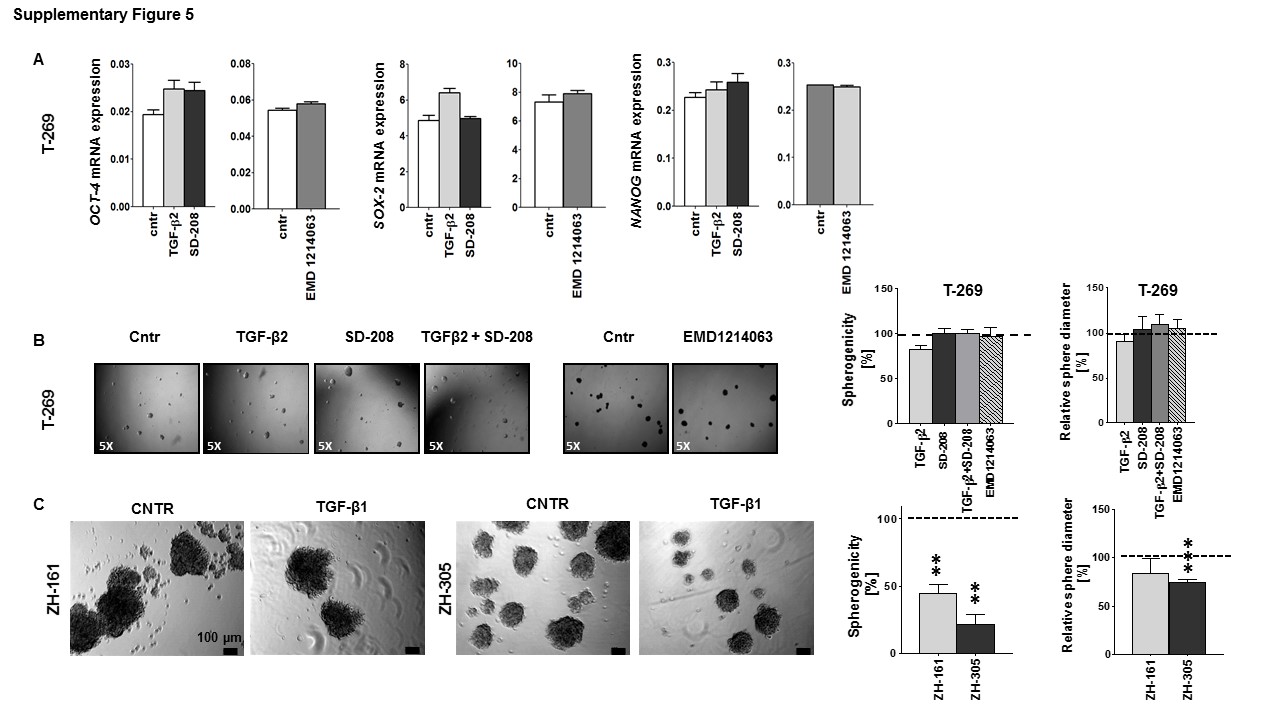

Supplement: Supplementary file 5 — Figure S5 [file 41419_2017_51_MOESM5_ESM.jpg]

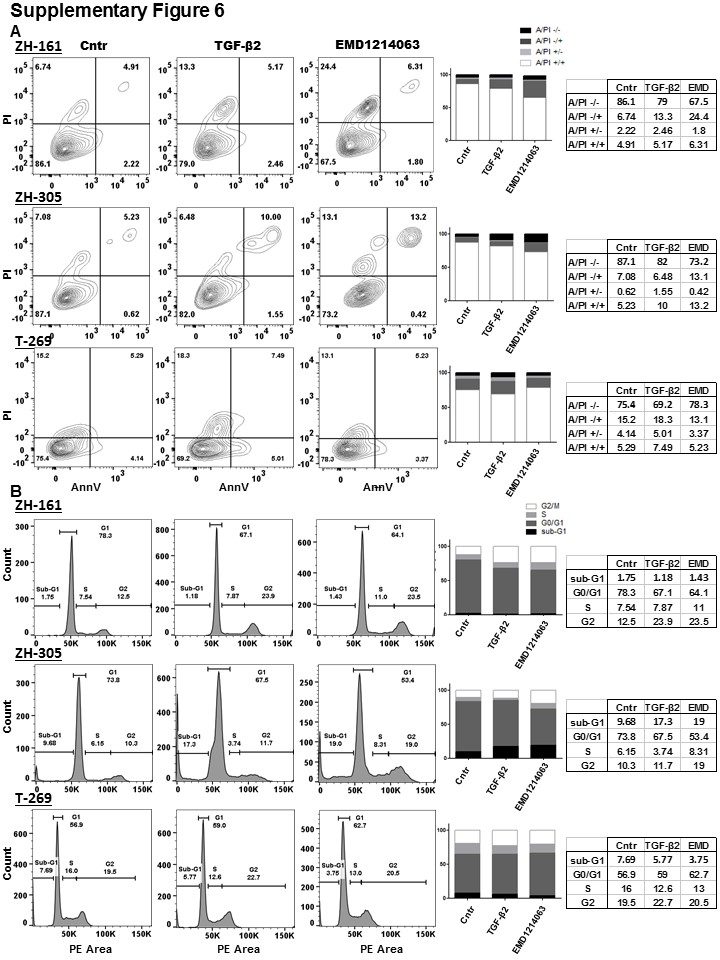

Supplement: Supplementary file 6 — Figure S6A,B [file 41419_2017_51_MOESM6_ESM.jpg]

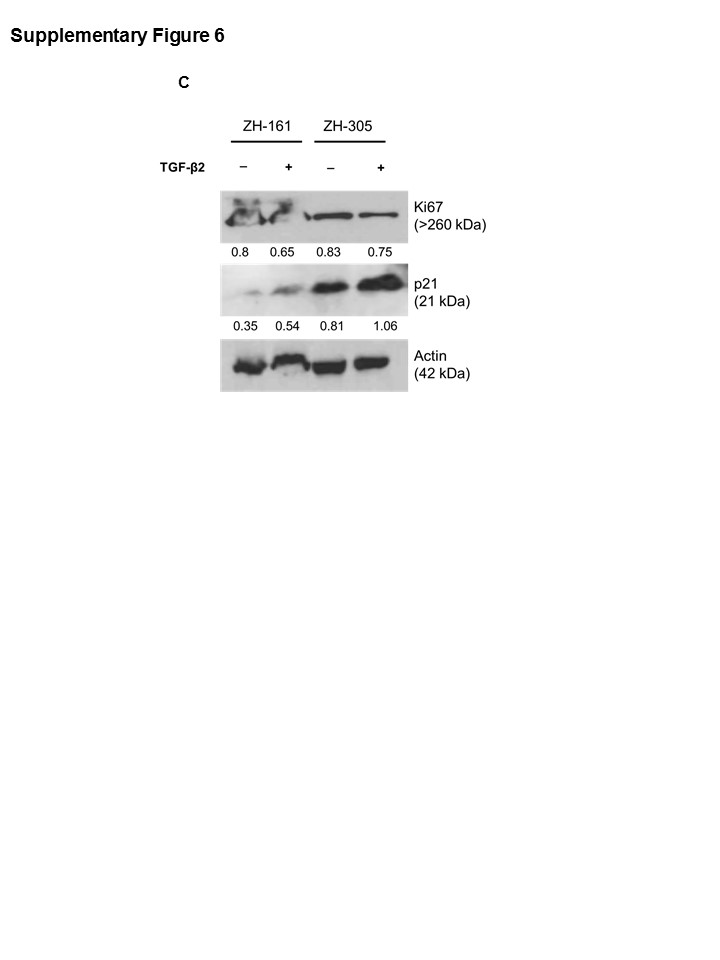

Supplement: Supplementary file 7 — Figure S6C [file 41419_2017_51_MOESM7_ESM.jpg]

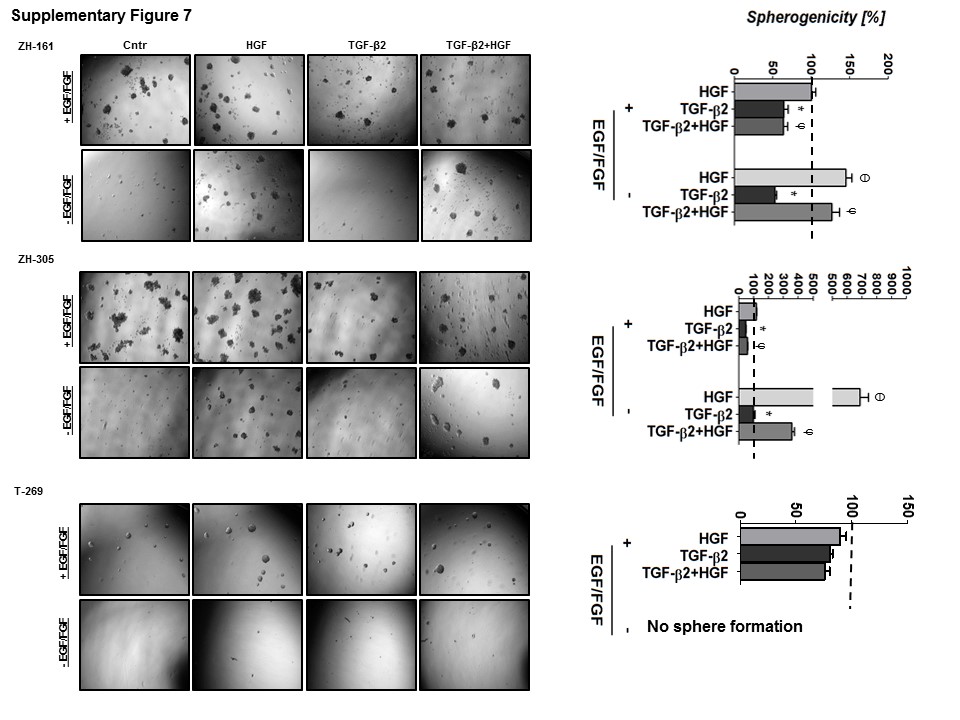

Supplement: Supplementary file 8 — Figure S7 [file 41419_2017_51_MOESM8_ESM.jpg]
