# Supplementary material for: Negative control of the HGF/c-MET pathway by TGF-β: a new look at the regulation of stemness in glioblastoma
Source: Cell Death Dis. 2017 Dec 13;8(12):3210. doi: 10.1038/s41419-017-0051-2 (PMC5870582; doi:10.1038/s41419-017-0051-2)
Supplement: Supplementary file 9 — Supplementary materials [file 41419_2017_51_MOESM9_ESM.docx]

**Supplementary materials**

**Supplementary Figure 1. TGF-β2 and p-c-MET antibody specificity studies.** Paraffin-embedded spheres were sectioned and stained for TGF-β2 (AP) (A) or p-c-MET (HRP) (B), using 0.5 µg/ml primary antibody or corresponding isotype controls (mouse and rabbit). Nuclei were counter-stained with hematoxylin (blue) (scale bars: 50 μm).

**Supplementary Figure 2. TGF-β1,2 and ALK-5 dependent control of the c-MET pathway.** A-C. ZH-161 cells were seeded in complete NB medium in the absence or presence of TGF-β1/TGF-β2 (2 ng/ml) (A), different concentrations of TGF-β2 (as indicated) (B), LY2157299 (1 µM) (C) and were assessed for c-MET/p-c-MET and TGF-β signaling molecules by immunoblot. D. Compared to the control siRNA transfected cells, 85% knockdown of *ALK-5* gene was achieved as assessed by RT-PCR. E. p-c-MET immunoreactivity upon TGF-β2 (2 ng/ml) treatment was assessed by immunocytochemistry. F. GIC were seeded in complete NB medium in the absence or presence of SD-208 (1 µM) or TGF-β2 (2 ng/ml) or both for 4 h. DMSO diluted in NB medium (1:20’000) served as a control. Modulation of *c-MET* mRNA expression in ZH-161 (left) or ZH-305 (right) cells was analyzed by RT-PCR (****p* < 0.001, effect of TGF-β2 compared to control, ^###^*p* < 0.001 effect of TGF-β2 and SD-208 co-treatment compared to TGF-β2 alone).

**Supplementary Figure 3. Role of SMAD in the control of c-MET activity by TGF-β2.** A-D. Transient gene ablation of SMAD2, SMAD3 or SMAD4 was performed by transfecting siRNA pools (100 nM final concentration). Where indicated, the cells were stimulated 2 h post-transfection with TGF-β2 (2 ng/ml) and assayed after 22 h in parallel with the unstimulated control cells. Control siRNA (100 nM) transfected cells were used as negative controls. Silencing efficiency of *SMAD* genes was assessed by RT-PCR (A) and immunoblot (B, C) in the absence or presence of TGF-β2. The effect of SMAD silencing on p-c-MET levels was also examined in ZH-161 (B) and ZH-305 (C) cells. Total c-MET and actin were assessed for reference. Expression levels of TGF-β target gene *PAI-1* were also quantified upon *SMAD* gene silencing (D).

**Supplementary Figure 4. Role of non-canonical TGF-β signaling components in the control of c-MET activity by TGF-β2.** A. p-ERK was measured in ZH-161 cells treated with U0126 (10 µM) or TGF-β2 (2 ng/ml) alone or in combination for 2 h in NB medium. p-SMAD2 was used as a positive control to confirm TGF-β2 activity. B,C. GIC were treated with TGF-β2 (2 ng/ml), U0126 (10 µM), AZD5363 (3 µM) alone or in combination under conditions described in Fig. 2 and *c-MET* mRNA levels were evaluated by RT-PCR (^*^*p* < 0.05, effect of TGF-β2 relative to control; ^ω^p< 0.05, effect of TGF-β2 and AZD5363 co-treatment relative to TGF-β2; ^ϴϴ^*p* < 0.001, effect of AZD5363 relative to control). D. Verification of the specificity of the 145-170 kDa bands and efficacy of c-MET gene silencing in c-MET positive GIC.

**Supplementary Figure 5. Modulation of stem cell marker expression and spherogenicity by TGF-β or c-MET pathways in GIC**. A. *OCT-4*, *SOX-2* or *NANOG* gene expression upon exposure to SD-208 (1 µM),TGF-β2 (2 ng/ml) or EMD1214063 (200 nM) (4 h) were assessed by RT-PCR. B. T-269 cells were plated at a density of 500 cells/well/100 µl. The cells treated with SD-208 (1 µM), TGF-β2 (10 ng/ml) or both or EMD1214063 (200 nM) were assessed for spherogenic capacity based on the number of spheres and average size at day 21 in triplicates. The photomicrographs were taken using a 5X objective. C. ZH-161 and ZH-305 cells were also assessed for changes in spherogenicity upon TGF-β1 treatment, ^**^*p* < 0.01 and ^***^*p* < 0.001, effect of TGF-β1 relative to control. The scale bars correspond to 100 μm.

**Supplementary Figure 6. Effect of TGF-β treatment and c-MET inhibition on viability and cell cycle progression.** A,B. ZH-161, ZH-305 and T-269 were exposed to TGF-β2 (2 ng/ml) or EMD1214063 (1 µM) for 72h. DMSO diluted in NB medium (1:20’000) served as a control. Induction of cell death was evaluated by annexin V (AnxV)/PI staining (A) and cell cycle phase distribution profiles were obtained by PI staining (B) using flow cytometry. Cell fractions are shown for non-treated *versus* treated cells in histograms as well as bar graphs. C. ZH-161 and ZH-305 were exposed to TGF-β2 (20 ng/ml) for 24h. Protein levels of Ki67 (proliferation marker) and p21 (inhibitor of cell cycle transition) were assessed by immunoblot. The relative quantification values are shown below the immunoblot panels.

**Supplementary Figure 7. Spherogenicity-promoting role of exogenous HGF.** ZH-161, ZH-305 or T-269 cells were seeded at a density of 500 cells/well/100 µl and grown for 21 days in HGF-containing (50 ng/ml) NB medium with (upper panels) or without EGF/FGF (lower panels). Stimulatory effects of HGF in the absence or presence of TGF-β2 (2 ng/ml) under these conditions were analyzed. Spherogenicity was determined by counting spheres. Sphere formation was assessed at day 21 in triplicates (^ϴ^*p* < 0.05, effect of HGF relative to control; ^*^*p* < 0.05, effect of TGF-β2 relative to control; ^φ^ < 0.05, effect of HGF and TGF-β2 co-treatment relative to TGF-β2).

**Table S1 List of primers and their corresponding sequences**

| **Gene** | **Forward sequence** | **Reverse sequence** |
| --- | --- | --- |
| ***HPRT1*** | **5'-TGAGGATTTGGAAAGGGTGT-3'** | **5'-GAGCACACAGAGGGCTAC AA-3'** |
| ***HGF*** | **5'-CCGAACAGGATTCTTTCACC-3'** | **5'-AGGAGGAGATGCAGGAGGAC 3'** |
| ***c-MET*** | **5'-ATGGAAATGCCTCTGGAGTG -3'** | **5'-CTGAGATACGCAGCCTGAAG-3'** |
| ***OCT-4*** | **5'-CGAGAAGGATGTGGTCCGAG-3'** | **5'-TGTGCATAGTCGCTGCTTGA-3'** |
| ***SOX-2*** | **5'-CACACTGCCCCTCTCAC-3'** | **5'-TCCATGCTGTTTCTTACTCTC-3'** |
| ***SOX-4*** | **5'-GGTCTCTAGTTCTTGCACGCT-3'** | **5'- CTGCAAGAAGGGAGCTGGTAA-3'** |
| ***MUSASHI*** | **5'-CCAATGGGTACCACTGAAGC -3'** | **5'-ACTCGTGGTCCTCAGTCAGC-3'** |
| ***CD133*** | **5'- TGGATGCAGAACTTGACAACGT -3'** | **5'-ATACCTGCTACGACAGTCGTGGT-3'** |
| ***PAI-1*** | **5'-CAGAAAGTGAAGATCGAGGTGAAC-3'** | **5'-GGAAGGGTCTGTCCATGATGAA-3'** |
| ***ALK-5*** | **5'-CTGGGAAATTGCTCGACGATG-3'** | **5'-ACTCTCAAGGCTTCACAGCTC-3'** |
| ***TGF-β1*** | **5'-GCCCTGGACACCAACTATTG-3'** | **5'-CGTGTCCAGGCTCCAAATG-3'** |
| ***TGF-β2*** | **5'-AAGCTTACACTGTCCCTGCTGC-3'** | **5'-TGTGGAGGTGCCATCAATACCT-3'** |
| ***TGF-β3*** | **5'-TCAGCCTCTCTCTGTCCACTT-3'** | **5'-CATCACCGTTGGCTCAGGG-3'** |
| ***SMAD2*** | **5'-GCACTTGCTCTGAAATTTGGGC-3'** | **5'-GACGACCATCAAGAGACCTGG-3'** |
| ***SMAD3*** | **5'-GCCTGTGCTGGAACATCATC-3'** | **5'-TTGCCCTCATGTGTGCTCTT-3'** |
| ***SMAD4*** | **5'-GGTTCCTTCAAGCTGCCCTA-3'** | **5'-ATGTGCAACCTTGCTCTCTCA-3'** |
| ***NANOG*** | **5'-GAAATACCTCAGCCTCCAGC-3'** | **5'-GCGTCACACCATTGCTATTC-3'** |
